# Gestational hypertension as a risk factor for increased postpartum hemorrhage volume in placenta previa: A retrospective study

**DOI:** 10.1097/MD.0000000000047731

**Published:** 2026-02-13

**Authors:** Hengyi Bai, Shan Chen, Yun Feng, Zhu Yang, Benfang Chen

**Affiliations:** a Department of Obstetrics, The Third Affiliated Hospital of Zunyi Medical University (The First People’s Hospital of Zunyi), Zunyi, Guizhou, China.

**Keywords:** gestational hypertension, placenta previa, postpartum hemorrhage, pregnancy, risk factors

## Abstract

This study aims to identify risk factors associated with postpartum hemorrhage (PPH) volume in pregnant women diagnosed with placenta previa. We retrospectively collected data on PPH volume in consecutive pregnant women at the First People’s Hospital of Zunyi between March 24, 2020, and February 28, 2024. The outcome variable, PPH volume, was divided into 5 categories: <500 mL, 500 to 1000 mL, 1001 to 1500 mL, 1501 to 2000 mL, and > 2000 mL, according to the Chinese Medical Association (CMA) obstetric guideline (2023 edition). These data were documented within 24 hours of birth. We examined maternal characteristics and concurrent pregnancy conditions to identify the potential risk factors for PPH volume. Univariate and multivariate ordered logistic regression analyses were used to determine the association between these factors and PPH volume, with the analysis conducted using SPSS statistical software (version 26.0). In total, 246 pregnant women were included in this retrospective study. Univariate analysis revealed that gestational hypertension increased the risk of an elevated volume of PPH, with an odds ratio (OR) of 5.336 (95% confidence interval [CI]: 1.204–23.656). This significance persisted in the multivariate ordered logistic regression analysis (OR = 6.445, 95% CI: 1.414–29.371), suggesting that pregnant women diagnosed with gestational hypertension are approximately 6.445 times more likely to experience a higher level of PPH volume than those without this condition. The mode of delivery, particularly cesarean section, was initially associated with a lower volume of PPH (OR = 0.393, 95% CI: 0.226–0.685); however, this association was not statistically significant in the multivariate analysis. Gestational hypertension significantly contributes to an increased PPH volume in patients with placenta previa. Clinicians must diligently monitor and manage such patients to mitigate the risk of severe PPH and related complications. Further research is required to validate our findings.

## 1. Introduction

Postpartum hemorrhage (PPH), defined as excessive blood loss following childbirth, remains a significant global health concern, accounting for approximately 11% of maternal deaths worldwide.^[1,2]^ Our study focuses on primary PPH, which refers to blood loss documented within 24 hours of birth.

Women with placenta previa, a condition in which the placenta attaches abnormally close to or over the cervix, are at an increased risk of PPH owing to the potential disruption of the placenta during delivery.^[3,4]^ While placenta previa itself is a strong independent risk factor for PPH, understanding the additional, modifying risk factors that contribute to the severity and volume of PPH in this already high-risk population is crucial for the development of effective prevention and management strategies.

Several factors are known to increase the risk of PPH in women with placenta previa, including advanced maternal age, high parity, mode of delivery, and various coexisting pregnancy conditions.^[5-7]^ Recent studies have explored the association between additional maternal characteristics and coexisting pregnancy conditions and PPH in women with placenta previa. For instance, a study found that a low maternal body mass index (BMI) was associated with an increased risk of PPH in women with placenta previa.^[4,8]^ Another study investigated the association between placental abruption and PPH in women with placenta previa.^[9]^

Although existing research has expanded our knowledge of PPH, there remains a notable scarcity of studies focusing on the risk factors that influence PPH volume. This gap highlights the need for a more comprehensive investigation, as using PPH volume (categorized as <500 mL, 500–1000 mL, 1001–1500 mL, 1501–2000 mL, and >2000 mL) allows for a more accurate assessment of risk factors compared to a simplified binary variable. Deeper insight into these factors could shape clinical approaches and ultimately improve outcomes for mothers and infants.This study aimed to investigate other potential risk factors associated with PPH volume in women with placenta previa. We explored the association between PPH volume and various maternal characteristics, coexisting pregnancy conditions, and newborn weight. Our findings have the potential to inform clinical practice and guide future research on PPH prevention and management in women with placenta previa.

## 2. Materials and methods

### 2.1. Subjects and study design

**Figure 1.**
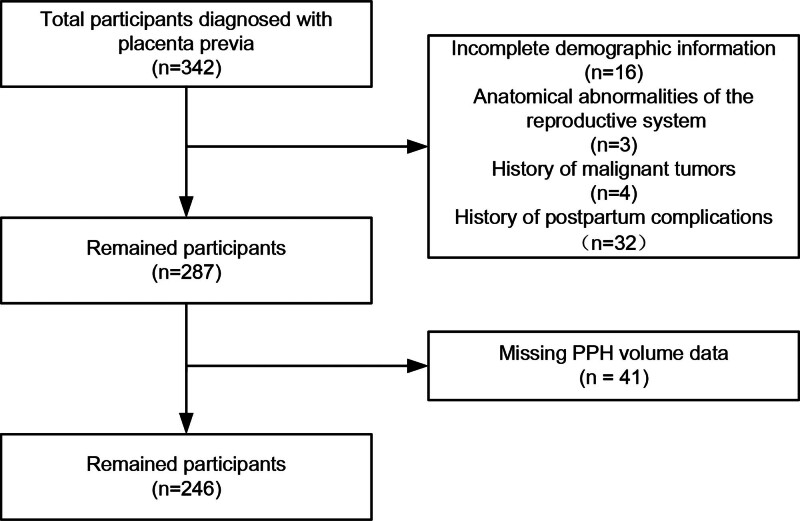
The flow chart of participant selection. PPH= postpartum hemorrhage.

We conducted a retrospective analysis of PPH volume, measured within 24 hours following childbirth, among consecutive pregnant women at the First People’s Hospital of Zunyi between March 24, 2020, and February 28, 2024. The necessary maternal, delivery, and outcome data were extracted from the hospital’s electronic medical records (EMR) system. Inclusion criteria were: diagnosis of placenta previa confirmed by ultrasonography; delivery at the First People’s Hospital of Zunyi between March 24, 2020 and February 28, 2024; and complete medical record data available. We excluded those with incomplete demographic or delivery details, anatomical reproductive system abnormalities, history of malignant tumors, or previous postpartum complications. Referral patients who delivered at our hospital were included if they met all other criteria. Of these, 342 were diagnosed with placenta previa. We excluded 96 patients (28.1%) due to incomplete demographic or delivery details, anatomical reproductive system abnormalities, history of malignant tumors, or previous postpartum complications. Ultimately, 246 pregnant women who delivered at our hospital and met all criteria were included in this study. Figure 1 shows the patient selection process used in this study. This study was approved by the Ethics Committee of First People’s Hospital of Zunyi (Approval Number: 20232-010). All procedures adhered to the principles of the Declaration of Helsinki. Patient data were retrospectively accessed and analyzed from electronic medical records. In accordance with hospital protocol, general consent for the use of anonymized medical records for research purposes had been obtained from all participants at the time of hospital admission, prior to study conception. Patient confidentiality was maintained throughout the study.

### 2.2. Data collection and definitions

Maternal characteristics, including age, gestational age, delivery methods, prepregnancy BMI, smoking history, history of alcohol consumption, gestational hypertension, preeclampsia, gestational diabetes, hypothyroidism, hyperthyroidism, and thrombocytopenia, were collected. Information on offspring birth weight was also included. PPH volume was recorded within 24 hours.

The PPH volume was categorized into 5 groups: <500 mL, 500 to 1000 mL, 1001 to 1500 mL, 1501 to 2000 mL, and more than 2000 mL. This categorization aligns with the severity grading of PPH defined by the Chinese Medical Association (CMA) obstetric guideline (2023 edition).^[10]^ PPH volume was recorded within 24 hours using a combination of gravimetric (weighing blood-soaked materials) and volumetric (using calibrated collection bags) methods, following established hospital protocol for Quantitative Blood Loss (QBL) estimation. All measurements were performed and documented by the operating physician or attending nurse.

Placenta Previa was defined as the condition in which the placenta attaches abnormally close to or over the cervix, confirmed by prenatal ultrasonography performed after 28 weeks of gestation and documented in the medical record. Prepregnancy BMI was calculated based on the individual’s weight and height using the formula BMI = Weight/(Height^2^). Delivery methods were classified as vaginal birth or cesarean section. Any smoking or alcohol consumption exceeding 3 instances during pregnancy was recorded as “Yes. ” This simple categorical cutoff was utilized due to the retrospective nature of the study and limitations in reliably documenting the precise frequency, quantity, or timing of exposure in the electronic medical records. This approach distinguishes patients with any reported exposure from those with zero reported exposure. Additionally, any accompanying diseases during pregnancy, such as gestational hypertension, preeclampsia, diabetes, hyperthyroidism, and hypothyroidism, were documented based on previous diagnoses made by attending physicians in the medical records according to established clinical guidelines. Platelet counts and hemoglobin levels were measured upon admission. Thrombocytopenia was diagnosed when the platelet count was less than 100 × 10^9^/L, and anemia was diagnosed when the hemoglobin level was <110 g/L. Moreover, in the case of twins, the recorded birth weight was the combined weight of both newborns. This combined weight was used as a single continuous variable in the analysis, which may reflect the confounding effect of uterine overdistension, a known risk factor for PPH.

### 2.3. Statistical analysis

All data analyses were conducted using SPSS statistical software (version 26.0; IBM Corp., Chicago). Categorical variables are expressed as frequencies and percentages, while continuous variables are expressed as mean ± standard deviation. Chi-square tests and Fisher’s exact test were used to compare categorical variables, and independent sample *t*-tests or the nonparametric Mann–Whitney *U* test were used to compare continuous variables. Statistical significance was set at *P* < .05.

Univariate logistic regression analysis was performed to assess the association between each factor (age, prepregnancy BMI, gestational weeks, delivery methods, smoking history, history of alcohol consumption, gestational hypertension, preeclampsia, twins, diabetes, anemia, hyperthyroidism, hypothyroidism, thrombocytopenia, and newborn weight) and PPH volume. This step helped to identify factors that had a potential association with PPH volume.

Multivariate ordered logistic regression analysis was performed to determine the independent factors significantly associated with PPH volume, while controlling for possible confounding factors. Variables significant at α = 0.20 from the univariate analysis were included in the multivariate ordered logistic regression model. The test of parallel lines was initially evaluated to ensure the proportional odds assumption was satisfied. If the *P*-value for test of parallel lines assumption test exceeded .05, the data were deemed suitable for multivariate ordered logistic regression (proportional odds model), thereby justifying the analysis.

In addition, the relationship between independent factors and PPH volume was further explored using logistic regression models.

## 3. Results

### 3.1. Baseline characteristics of the study population

Baseline characteristics of the study population are presented in Table 1. There were no significant differences in age (*P* = .271), prepregnancy BMI (*P* = .422), or gestational weeks at delivery between the different PPH volume groups. However, the delivery method showed a significant association with PPH volume (*P* < .001), with a higher proportion of cesarean sections observed in the groups with greater blood loss. Additionally, the presence of gestational hypertension was found to be significantly associated with PPH volume (*P* = .049), whereas other medical conditions and newborn weight did not show significant associations. These findings highlight the potential impact of delivery methods and gestational hypertension on PPH volume, and provide insights for further investigation.

**Table 1:** Patient demographics and baseline characteristics.

Characteristic	PPH volume					*P*-value
	<500, N = 95*	500–1000, N = 112*	1001–1500, N = 22*	1501–2000, N = 5*	>2000, N = 11*	
Age	30.7 ± 4.6	30.1 ± 5.0	30.0 ± 4.7	28.8 ± 3.0	33.1 ± 3.9	.271†
Prepregnancy BMI	22.3 ± 3.4	21.8 ± 2.7	21.4 ± 2.6	22.5 ± 4.1	23.0 ± 5.6	.422†
Gestational wk						
27–36	45 (47.4%)	41 (36.6%)	5 (22.7%)	1 (20.0%)	6 (54.5%)	
37–38	38 (40.0%)	28 (25.0%)	9 (40.9%)	0 (0.0%)	3 (27.3%)	
39–41	12 (12.6%)	43 (38.4%)	8 (36.4%)	4 (80.0%)	2 (18.2%)	
Delivery methods						<.001‡
Natural birth	9 (9.5%)	43 (38.4%)	6 (27.3%)	2 (40.0%)	2 (18.2%)	
Cesarean section	86 (90.5%)	69 (61.6%)	16 (72.7%)	3 (60.0%)	9 (81.8%)	
Smoking history						>.999‡
No	95 (100.0%)	111 (99.1%)	22 (100.0%)	5 (100.0%)	11 (100.0%)	
Yes	0 (0.0%)	1 (0.9%)	0 (0.0%)	0 (0.0%)	0 (0.0%)	
History of alcohol consumption						>.999‡
No	94 (98.9%)	111 (99.1%)	22 (100.0%)	5 (100.0%)	11 (100.0%)	
Yes	1 (1.1%)	1 (0.9%)	0 (0.0%)	0 (0.0%)	0 (0.0%)	
Gestational hypertension						.049‡
No	94 (98.9%)	110 (98.2%)	21 (95.5%)	4 (80.0%)	10 (90.9%)	
Yes	1 (1.1%)	2 (1.8%)	1 (4.5%)	1 (20.0%)	1 (9.1%)	
Preeclampsia						>.999‡
No	94 (98.9%)	110 (98.2%)	22 (100.0%)	5 (100.0%)	11 (100.0%)	
Yes	1 (1.1%)	2 (1.8%)	0 (0.0%)	0 (0.0%)	0 (0.0%)	
Twins						.453‡
No	95 (100.0%)	109 (97.3%)	22 (100.0%)	5 (100.0%)	11 (100.0%)	
Yes	0 (0.0%)	3 (2.7%)	0 (0.0%)	0 (0.0%)	0 (0.0%)	
Diabetes						.873‡
No	82 (86.3%)	92 (82.1%)	19 (86.4%)	5 (100.0%)	10 (90.9%)	
Yes	13 (13.7%)	20 (17.9%)	3 (13.6%)	0 (0.0%)	1 (9.1%)	
Anemia						.282‡
No	79 (83.2%)	101 (90.2%)	20 (90.9%)	5 (100.0%)	8 (72.7%)	
Yes	16 (16.8%)	11 (9.8%)	2 (9.1%)	0 (0.0%)	3 (27.3%)	
Hyperthyroidism						.468‡
No	93 (97.9%)	111 (99.1%)	21 (95.5%)	5 (100.0%)	11 (100.0%)	
Yes	2 (2.1%)	1 (0.9%)	1 (4.5%)	0 (0.0%)	0 (0.0%)	
Hypothyroidism						.133‡
No	89 (93.7%)	106 (94.6%)	18 (81.8%)	4 (80.0%)	10 (90.9%)	
Yes	6 (6.3%)	6 (5.4%)	4 (18.2%)	1 (20.0%)	1 (9.1%)	
Thrombocytopenia						.252‡
No	92 (96.8%)	106 (94.6%)	21 (95.5%)	4 (80.0%)	10 (90.9%)	
Yes	3 (3.2%)	6 (5.4%)	1 (4.5%)	1 (20.0%)	1 (9.1%)	
Weight of the newborn						.184‡
1450–2899	38 (40.0%)	32 (28.6%)	4 (18.2%)	2 (40.0%)	7 (63.6%)	
2900–3399	33 (34.7%)	40 (35.7%)	11 (50.0%)	2 (40.0%)	2 (18.2%)	
3400–6100	24 (25.3%)	40 (35.7%)	7 (31.8%)	1 (20.0%)	2 (18.2%)	

BMI = body mass index, PPH = postpartum hemorrhage.

* Mean ± SD; n (%).

† One-way ANOVA.

‡ Fisher’s exact test.

### 3.2. Gestational hypertension serves as a risk factor for postpartum hemorrhage volume

In univariate analysis (Table 2), gestational hypertension emerged as a significant risk factor for increased PPH volume (odds ratio [OR] = 5.336, 95% CI [1.204–23.656], *P* = .028). Notably, a strong association was found between delivery method and postpartum blood loss; specifically, cesarean section was associated with a reduced risk of PPH (OR = 0.393, 95% CI [0.226–0.685], *P* = .001). No significant associations were found between PPH volume and other factors, such as age, prepregnancy BMI, smoking history, and history of alcohol consumption. The variables included in the multivariate ordered logistic regression, with a significance level of *P* < .2, were gestational weeks, delivery methods, gestational hypertension, and hypothyroidism.

**Table 2:** Univariate logistic regression analysis of PPH volume based on clinical data.

Characteristic	Total (N)	Estimate	OR (95% CI)	*P*-value
Age	245	−0.007	0.993 (0.945–1.044)	.782
Prepregnancy BMI	245	−0.038	0.962 (0.893–1.037)	.312
Gestational wk				
39–41	69	0.988	2.687 (1.481–4.875)	**.001**
37–38	78	−0.048	0.953 (0.540–1.681)	.867
27–36 (reference)	98	1.0	1.0	
Delivery methods				
Cesarean section	183	−0.934	0.393 (0.226–0.685)	**.001**
Natural birth (reference)	62	1.0	1.0	
Smoking history				
Yes	1	0.623	1.864 (0.048–72.883)	.739
No (reference)	244	1.0	1.0	
History of alcohol consumption				
Yes	2	−0.640	0.527 (0.035–7.947)	.644
No (reference)	243	1.0	1.0	
Gestational hypertension				
Yes	6	1.674	5.336 (1.204–23.656)	**.028**
No (reference)	239	1.0	1.0	
Preeclampsia				
Yes	3	−0.153	0.858 (0.100–7.393)	.889
No (reference)	242	1.0	1.0	
Twins				
Yes	3	0.630	1.878 (0.224–15.753)	.561
No (reference)	242	1.0	1.0	
Diabetes				
Yes	37	−0.001	0.999 (0.517–1.930)	.997
No (reference)	208	1.0	1.0	
Anemia				
Yes	32	−0.385	0.680 (0.335–1.382)	.287
No (reference)	213	1.0	1.0	
Hyperthyroidism				
Yes	4	−0.210	0.811 (0.125–5.275)	.826
No (reference)	241	1.0	1.0	
Hypothyroidism				
Yes	18	0.618	1.855 (0.754–4.569)	.179
No (reference	227	1.0	1.0	
Thrombocytopenia				
Yes	12	0.690	1.993 (0.672–5.911)	.213
No (reference)	233	1.0	1.0	
Weight of the newborn (g)				
3400–6100	74	0.324	1.382 (0.765–2.500)	.284
2900–3399	88	0.248	1.282 (0.727–2.260)	.391
1450–2899 (reference)	83	1.0	1.0	

Key textual explanations for bold values (*P* < .05, statistically significant).

BMI = body mass index, CI = confidence interval, OR = odds ratio, PPH = postpartum hemorrhage.

The parallelism assumption test yielded a *P*-value of .662, indicating that multivariate ordered logistic regression analysis is suitable for further investigation in this study. In the multivariate ordered logistic regression analysis (Table 3), after adjustment for potential confounders, gestational hypertension remained significantly associated with PPH volume (OR = 6.445, 95% CI [1.414–29.371], *P* = .016), suggesting that pregnant women diagnosed with gestational hypertension were approximately 6.445 times more likely to experience a higher level of PPH volume than those without this disease. However, the wide confidence interval (CI) for this odds ratio suggests limited precision, which is likely a consequence of the small sample size for patients with gestational hypertension. Moreover, the association between the method of delivery and PPH volume was not significant in multivariate analysis (OR = 0.613, 95% CI [0.304–1.236], *P* = .172). Hypothyroidism was also introduced in the model but was not found to be significantly associated with PPH volume (OR = 1.510, 95% CI [0.604–3.776], *P* = .379).

**Table 3:** Multivariate ordered logistic regression analysis of PPH volume.

Parameter	Characteristic	Estimate	*P*-value	OR (95% CI)
Threshold	[PPH volume = 1]	−0.583	.145	0.558 (0.255–1.223)
	[PPH volume = 2]	1.741	.000	5.70 (2.518–12.922)
	[PPH volume = 3]	2.756	.000	15.730 (6.372–38.827)
	[PPH volume = 4]	3.164	.000	23.663 (9.013–62.123)
Location	Gestational wk			
	39–41	0.695	.069	2.003 (0.948–4.223)
	37–38	−0.032	.914	0.969 (0.544–1.724)
	27–36 (reference)	1.0		1.0
	Delivery methods			
	Cesarean section	−0.489	.172	0.613 (0.304–1.236)
	Natural birth (reference)	1.0		1.0
	Gestational hypertension			
	Yes	1.863	.016	**6.445 (1.414–29.371**)
	No (reference)	1.0		1.0
	Hypothyroidism			
	Yes	0.412	.379	1.510 (0.604–3.776)
	No (reference)	1.0		1.0

Key textual explanations for bold values (*P* < .05, statistically significant).

CI = confidence interval, OR = odds ratio, PPH = postpartum hemorrhage.

### 3.3. Sensitivity analysis

A sensitivity analysis was performed to explore the association between gestational hypertension and PPH volume. As shown in Table 4, gestational hypertension was significantly associated with PPH volume in all 3 models. In model 1, in which no covariable was adjusted, gestational hypertension was found to significantly increase the odds of PPH volume (OR = 5.336, 95% CI [1.204–23.656], *P* = .028). This association remained significant even after adjusting for age and prepregnancy BMI in model 2 (OR = 5.106, 95% CI [1.148–22.707], *P* = .032) and further adjusting for weeks of gestation, delivery methods, smoking history, history of alcohol consumption, preeclampsia, twins, diabetes, anemia, hyperthyroidism, hypothyroidism, thrombocytopenia, and weight of the newborn in model 3 (OR = 5.917, 95% CI [1.194–29.320], *P* = .029).

**Table 4:** Association between gestational hypertension and PPH volume (multinomial ordered logistic regression).

Characteristic	Model 1			Model 2			Model 3		
	OR1	95% CI1	*P*-value	OR2	95% CI2	*P* -value	OR3	95% CI3	*P* -value
Gestational hypertension (no)	1.00	1.00, 1.00	.028	1.00	1.00, 1.00	.032	1.00	1.00, 1.00	.029
Gestational hypertension (yes)	5.336	1.204, 23.656	.028	5.106	1.148, 22.707	.032	5.917	1.194, 29.320	.029

Model 1: no covariable adjusted.

Model 2: adjusted for age and prepregnancy BMI.

Model 3: adjusted for age, prepregnancy BMI, gestational weeks, delivery methods, Smoking history, alcohol consumption history, preeclampsia, twins, diabetes, anemia, hyperthyroidis, hypothyroidism, thrombocytopenia and weight of the newborn.

BMI = body mass index, CI = confidence interval, OR = odds ratio, PPH = postpartum hemorrhage.

## 4. Discussion

In this study, we investigated the risk factors associated with PPH volume in women with placenta previa. Our primary finding was that gestational hypertension was a significant and independent risk factor for increased PPH volume. Conversely, cesarean section delivery was associated with a lower risk of PPH than vaginal birth, although this association was not statistically significant in the multivariate analysis. Other investigated factors, including maternal characteristics, coexisting pregnancy conditions, and newborn weight, did not exhibit statistically significant associations with PPH volume.

Consistent with our findings, previous research has shown a lower risk of PPH with cesarean section delivery than with vaginal birth. One study reported a PPH incidence of 57.7% for vaginal deliveries versus 28.2% for cesarean sections when using a blood loss threshold ≥ 500 mL threshold.^[11]^ A cohort study concluded that cesarean section was associated with a significantly lower risk of maternal mortality, maternal hemorrhage, need for blood transfusion, and peripartum hysterectomy than vaginal delivery in women with placenta previa.^[12]^ This protective effect is likely due to controlled delivery and reduced risk of lacerations and uterine atony, which are common causes of PPH during vaginal birth.

Similarly, gestational hypertension has been established as a risk factor for PPH in prior studies. Studies have shown that women with gestational hypertension are more likely to experience PPH compared to those without hypertension during pregnancy.^[9,13,14]^ A meta-analysis of 14 studies reported that women with gestational hypertension had a significantly higher risk of PPH than those without hypertension during pregnancy.^[13]^ The pooled OR was 6.08 (95% CI: 3.67–10.08), indicating over a 6-fold increased risk of PPH in women with gestational hypertension.^[13]^ One cohort study conducted across 38 hospitals in the Netherlands developed predictive models to assess the risk of PHH in women with gestational hypertension or mild preeclampsia, confirming these conditions as significant risk factors.^[9]^ These findings suggest that gestational hypertension is a consistent risk factor of PPH across various populations and study designs.

In our study, we utilized multivariate ordered logistic regression analysis, which offers 2 key advantages. First, it permits simultaneous evaluation of the relationships of multiple factors with PPH volume while controlling for potential confounders. Second, it captures the nuances of changes in PPH volume, an ordered multicategorical variable. In contrast to previous studies that simplified PPH volume into a binary variable, our method allows for a more accurate assessment of risk factors, providing precise information for clinical decision-making.

Several mechanisms may explain the association between gestational hypertension and PPH. Gestational hypertension may lead to vascular dysfunction characterized by impaired endothelial function and increased vascular permeability.^[15,16]^ This dysfunction can cause excessive bleeding during delivery due to fragile blood vessels and impaired platelet aggregation.^[17,18]^ Additionally, gestational hypertension may be associated with a higher prevalence of maternal morbidities, such as preeclampsia and placental abruption, which further increase the risk of PPH.^[9,19,20]^ Our study strengthens existing evidence by providing further confirmation of the link between gestational hypertension and PPH volume in women with placenta previa. Our findings highlight the importance of considering gestational hypertension as a risk factor for PPH in clinical practice.

Our study has some limitations. First, the retrospective nature of our study design limits the ability to establish causal relationships. In addition, the single-center design restricts the generalizability of our findings to other populations. Moreover, the relatively small sample size may have limited our ability to detect associations with weaker effect sizes. Future research with a prospective design, larger sample sizes, and multicenter cohorts is necessary to address these limitations and to strengthen the evidence base.

## 5. Conclusion

Gestational hypertension is a significant risk factor for an increased PPH volume in women with placenta previa. Conversely, while Cesarean section was initially associated with a lower volume of PPH in univariate analysis, this association was not statistically significant in the multivariate model. Clinicians should be vigilant in monitoring and managing women with placenta previa and gestational hypertension to minimize the risk of severe PPH and its associated complications. Further research is warranted to elucidate the underlying mechanisms and to develop effective prevention and treatment strategies for PPH in this high-risk population.

## Acknowledgments

We thank Jinlong Huo for providing the text-refinement service.

## Author contributions

**Conceptualization:** Zhu Yang, Benfang Chen.

**Data curation:** Hengyi Bai, Shan Chen.

**Formal analysis:** Yun Feng.

**Software:** Yun Feng.

**Writing – original draft:** Hengyi Bai, Zhu Yang, Benfang Chen.

**Writing – review & editing:** Zhu Yang, Benfang Chen.
